# Hermaphrodism, from a Medico-Legal Point of View

**Published:** 1875-12

**Authors:** 


### Translations.

### HERMAPHRODISM,

### FROM A MEDICO-LEGAL POINT OF VIEW.

*Translated from the French of Basile Poppesco,*

By E. WARREN SAWYER, M.D.,

Lecturer on Obstetrics, Rush Medical College, Chicago.

(Concluded from page 876.)

### OBSERVATIONS.

Observation I, (due to the kindness of Dr. Châtillon). Apparent hermaphrodism in a person of the male sex.

P——, aged nineteen years, had resided in Paris since he was five years old. A consultation had been held a long time previously, at which MM. Ricord and Clerc were present. He wishes now to know if he can, or cannot receive a *carte*, which he claims, in order that he may give himself more fully to prostitution. It was decided that this *carte* should be refused; because, P—— was a man.

He was considered as the subject of complete hypospadias, with this peculiarity, that the penis was so rudimentary that its volume did not exceed that of a well developed clitoris. The urethral canal was larger than normal, but it terminated in the bladder and not in a vagina. The rectal touch did not permit one to feel an uterus, nor had menstruation been established. However, the general configuration of the body, the aspect of the face, the quality of the voice, are rather those of the woman; the breasts, moreover, are quite rudimentary; the skin is smooth in those places where it normally is in the woman.

Concerning the tastes of P——, they appear to be those of a woman, and he denies with energy, and angrily, the accusation made against him, which is that he has a predilection for women.

In this instance, despite the external appearances, the tastes and the feminine aptitudes, one ought to conclude that this is simply a case of arrest of development of the sexual parts, accompanied by cryptorchidia.

Obs. II. Apparent hermaphrodism in an individual of the male sex.

In the month of December, 1873, there entered la Pitiè, hall Saint-Jean, in the service of M. Labbé, a person presenting one of the most remarkable instances of hermaphrodism. This account may appear a little too imperfect; but I was only able to procure the following details, which, however, are much the most important.

In November, 1873, a young girl of about seventeen years, living in Asnières, went to consult a physician for a crural hernia. While examining this hernia, he was much surprised to find a penis as voluminous as that of the majority of young men of that age. The penis had a prepuce which could be made to cover it entirely, but, from habit, the penis was uncovered almost to the corona glandis; it presented no trace of the canal of the urethra. On the lateral parts, one could not recognize either the labia majora or nymphæ, not even the slightest fold which could be considered rudimentary. But, between the penis and the anus, almost equi-distant from both, there was an oval opening, which allowed with difficulty the passage of the little finger, and which could be penetrated but a little way, because of the violent pains which were provoked by the attempt. The patient urinated through this oval orifice. It has been impossible to ascertain if, at the extremity of this canal, the uterus could be found, on account of the narrowness of the parts: the rectal touch even, in this connection, has only furnished negative information.

This pretended young girl had never had her catamenia; her features were rather those of a man than of a woman; she had no beard, but the loud voice was masculine; the breasts were markedly developed.

The presence of the testicles could not be established. The person admitted that she experienced a great pleasure when she was with the young girls, and seems to have felt, on several occasions, when touched by them, some voluptuous sensations, but has never had manifest ejaculations.

The conformation of the penis, the presence of the glans enveloped by a prepuce, the absence of the labia majora and nymphæ, but especially the negative results obtained from the search for the uterus and ovaries, and the absence of the multiple facts which could make known a menstrual elaboration, permit us to conclude, especially in view of the external physical characteristics, that this pretended young girl has been, to this time, incorrectly entered on the registers of civil state as being of the female sex, but is, in reality, a male, in whom the development of the external genital parts presents a malformation well known in science.

Obs. III, (due to the extreme kindness of my learned master, M. Gueneau de Mussy). Apparent hermaphrodism in the male sex.

In 1848, I was consulted by a woman for her daughter, who, eleven years of age, had for some time exhibited many disturbances of her health. I was immediately struck by the walk of this child; there was something masculine in her physiognomy, and in her manner; the volume of the chest was noticeable at the base; supposing a malformation, I demanded of the mother if her child presented nothing peculiar; she responded to me, blushing, that there was something extraordinary in her conformation, but, till then, she had preserved the most absolute secrecy respecting this. At my solicitation, the mother submitted her child to my examination, and this is what I recognized. The pubis was covered with hair, and had been since the age of five, according to the mother. Beneath the pubis, there existed a sort of penis, having a length of about two-thirds of an inch, and terminating in a slightly swollen extremity; on its inferior surface, there was a groove, representing the superior wall of the urethra, continuing beyond at the level of the orifice of a veritable canal, which opened behind the base of the penis. Still posteriorly, there existed a kind of vulva, bordered by two rudimentary labia; this vulva limited inferiorly a little vaginal canal, into which I could introduce my little finger, and which terminated superiorly in a *cul-de-sac*. There was no trace of the testicles, either in the labia, or at the level of the rings. Having introduced a sound into the bladder, and my index finger into the rectum, I was enabled to recognize that my finger was separated from the sound only by a membrane, and consequently I was able to conclude, with approximative certainty, that the uterus did not exist.

The principal details of this observation permit us thus to affirm the existence of a masculine sexuality, with arrest of development, which had an apparent similarity of the external genital organs to those of the female sex.

Obs. IV. Hermaphrodism with sex imperfectly defined. (Personal observation).

In 1867, when I was physician of the District of ——, I was asked by one of my countrymen to attend one of his friends. Upon reaching the place, I found myself in the presence of a young man, attired as a young girl, of about twenty-five years of age, offering a remarkable physiognomy, encircled by a head of long hair, and presenting not the slightest trace of moustache or whiskers. There was a slight something in the manners and carriage of the person, such as led me to demand unexpectedly if he was a man or a woman.

In informing me of his trouble, he began, blushing, by praying me to preserve with the greatest secrecy the details which I should observe. Then he passed into his chamber, where some minutes after I found him in bed.

This chamber was magnificently ornamented ; innumerable perfumes were scattered here and there ; everything, in a word, in the apartment, revealed the most effeminate coquetry.

He told me that he suffered in the genital parts ; when I proceeded to a minute and attentive examination of these organs.

The clitoris was excessively developed, having a length of an inch and two-thirds, or two inches ; at its summit it terminated in an enlarged extremity, quite suggestive of the glans ; this organ was imperforate. Beneath, there was a little opening through which the patient urinated, leading into a cavity which admitted the finger without any difficulty. Nevertheless, it was impossible for me to feel the neck of the uterus, and there was nothing in the antecedents which showed a more or less clearly defined menstrual elaboration. The labia majora were normally formed. On the internal surface of the right labium, there was a soft chancre, which had been communicated by the gentleman who had solicited my attendance, and who, I was told, was the lover (l'amant) of the patient. The pubis was covered with hair. Nowhere was there any trace of the testicles. The thorax was large, but the breasts were scarcely developed ; the voice was perfectly feminine.

This person had not the slightest predilection for women. Being possessed of a great fortune, he retained in his service a *personnel* of male domestics.

Subsequently, on several occasions, I saw him dressed in the most fantastic costumes, in garments of brilliant colors. At home he always remained clothed as a woman, but when he went out, he wore male garments.

In this observation, the positive conclusion seems very difficult, and we are inclined to offer the opinion that this is a instance of hermaphrodism in which it is impossible to define the sex. The absence of the testicles, notwithstanding the abnormal development of the penis, forces one to question a masculine sexuality, while the absence of all menstruation, the absence of the uterus and ovaries, prevents us from concluding, in a positive manner, upon a feminine sexuality.

Obs. V. Bisexual hermaphrodism.

Dr. Ceccherelli published in the journal *Lo Sperimentale*, of February, 1874, (Florence), the description of an hermaphrodite whom he had been called to examine. The subject had already been studied by Rokitansky, Seultz, and especially by Virchow, who had published a description of him in his *Archives of Pathological Anatomy*.

This monster is forty years old. The breasts are largely developed and pendulous ; the right eye is larger than the left. The penis at the meatus has hypospadias ; but a sound can be entered, which reveals another opening, two-thirds of an inch from the meatus, through which the urine and spermatic fluid is passed. There is but one, very well developed testicle. The penis and scrotum on the right, and the prepuce on the left, is the arrangement which, till recently, had been mistaken for a vulva, and the person had been considered as belonging to the female sex, and had received the name of Catherine. The glans had been mistaken for the clitoris. In introducing the finger between the two folds, below the glans, an eminence was felt, having all the characters of the neck of the uterus. (Seultz).

The female apparatus is complete, and is situated behind a little septum formed by the skin.

Bilroth had proposed the incision of this septum, in order to make more evident the characters of the female sex, which are more in keeping with all the other signs of the hermaphrodism. The catamenia have always been regular till within the past two years. He has, moreover, accomplished the function of copulation of the female sex. The prostate cannot be reached, on account of the uterus. The vesiculæ seminales exist, without doubt, for Catherine is able to be, in turn, the active party of coition, and the sperm, which has been examined by Virchow, contains spermatozoids. (Prog. Méd., 1874.)

This observation appears to us, to be, notwithstanding the brevity of the details which have been given to us, one of the clearest examples of bisexual hermaphrodism: and from a medico-legal point of view, it seems to us that it will be very difficult to establish the civil rank of this person, for the female sexuality appears, at least, as evident as the male sexuality. But, in view of similar cases, considering especially the positive results furnished by the examination of the sperm, and further, on the contrary, that utero-ovarian fecundation will be impossible on account of the abnormal septum, we would not hesitate, under parallel circumstances, to pronounce in favor of a male sexuality, and to inscribe as males, on the registers of civil state, all persons who exhibit the same vices of conformation.

Obs. VI. Bisexual hermaphrodism. By M. Odin, Interne of the Hospitals.*

Nat. Per., day laborer, aged sixty-three years, born at Millery, (Rhône), entered the Hôtel-Dieu, of Lyon, June 10, 1873, in a state of absolute coma, resulting from an attack of apoplexy on that day, and died on the fourteenth of the same month without regaining his consciousness.

The autopsy made on the following day, under the direction of Dr. Bondet, showed traces of an abundant cerebral hæmorrhage, which had caused the coma and death.

The subject is of medium height; body considerably emaciated; the extremities are short, slim and rounded; the hands and feet are small; the skin fine. The pelvis is narrow and contracted; the hips approach each other; in a word, it has the masculine conformation.

There is but a slight growth of hair, though the scalp and pubis are well covered, while the axillæ have scarcely any hair; the remainder of the surface is bare, and the surroundings of the anus entirely so.

He has no breasts, but the nipples are well developed.

The genital organs present: first, a penis surmounted by a glans; this is imperforate; it has a corona at its base and a furrow on its inferior surface. It is covered with a prepuce throughout its entire extent, save at the inferior part at the level of the furrow.

The penis, measured on the cadaver, was three and one-third inches in length, and nearly of the normal size. Upon its inferior surface, there was a furrow, continuous with that at the glans which termiated in an orifice, situated below and in front of the pubis, through which the urine flowed. The penis is inserted at the commissure of the labia majora which gives origin to it, being continuous with the skin that covers it like a hood, resembling the clitoris.

The scrotum is cleft, and has the aspect of the labia majora covered with hair on their exterior, but smooth, having a semi-mucous surface, on their internal surface. They terminate in front in uniting at the root of the penis, and inferiorly by gradually becoming thinner, and without uniting to form a *fourchette.*

At the bottom of the space included between the labia majora, is the median raphe of the perineum, which extends from the

* Lyon Médical, No. 13 (21 Juin, 1874.)

urinary orifice to the anus, and measures two and one-third inches.

On the right side, near the inguinal ring, there is a little rounded tumor, of the size of a pigeon's egg, which contains a little body, round and movable.

On the other side, the ring has no tumor analogous to the preceding, it is entirely free.

In catherizing the urinary orifice, one arrives at very different results according as he follows the anterior or posterior wall of the canal. In following the first, the bladder is reached; but in following the second, one reaches a very deep cavity situated behind the bladder.

From the external examination, it is shown that we have to do with an hermaphrodite belonging to the male sex, from the presence of a penis a little irregular, a testicle, and the absence of the breasts and the form of the pelvis ; and to the female sex, by the presence of the labia majora, the slight development of the hair upon the surface, the general aspect of the body, and the existence of a cavity behind the bladder, revealed by the catheter.

Notwithstanding these numerous similarities to the female sex, it seems more rational to assign this person to the male sex, to which he belonged during his life.

The internal generative organs are still less characteristic of the sex of the person.

Beginning at the orifice, which we have described, there is a canal of uniform calibre, an inch and a third in length, and one-third of an inch in diameter, lined with mucous membrane which is continuous with that of the labia majora ; it terminates posteriorly by an orifice completely closed by an annular membrane like a hymen.

Anterior to this hymen, on the anterior wall of this canal, there is a smaller orifice which leads into the bladder ; this is the true meatus urinarius.

This canal is quite anomalous, it being neither the urethra, though there is no other, nor the vagina, it being anterior to the hymen. It is a canal common to the urine, and the secretions from the vagina, formed by the two nymphæ and representing the vulva.

If we go beyond the orifices which we have described, by the anterior, the bladder can be reached, through a canal two-thirds of an inch long. The prostate is entirely wanting. By the posterior orifice, the vagina is reached, having a length of about three inches and a circumference of two inches. The mucous membrane lining the vagina is smooth, and has no trace of columns. There is a little mucus in this cavity. At the superior part, there is a slight constriction, which is directly continuous with the lips of the cervix uteri, without forming a *cul-de-sac*.

The uterus has a large, rounded neck; its walls are thin, with a well marked arbor vitæ.

The cervix is not sensibly distinct from the body.

The body is rudimentary; it diminishes in size from the neck towards the fundus, becomes rounded and terminates in a long cord, of the size of a goose quill, and inclined to the left. The cavity of the uterus is two and two-thirds inches long; it terminates in a *cul de-sac*. The uterus is received into a fold of peritoneum, which has the position, the direction and the form of the great ligaments. It even has the three alæ, but slightly marked.

In following the course of the cord in which the uterus terminates, a fleshy mass is reached, situated at the peritoneal orifice of the inguinal canal, making a prominence on the peritoneal side. It is enveloped in a fold of peritoneum, which forms a tunic, analogous to the tunica vaginalis. In this mass there is a partially developed testicle, surmounted by a voluminous epididymis; an imperforate Fallopian tube, with a well formed pavilion, and the fimbriated extremity directed towards an elongated irregular body, covered with little swellings, and even presenting little cysts. This body is probably the ovary, which it resembles, though the microscope has not revealed the presence of ovules. Between the Fallopian tube and the ovary, there is a rudimentary body of Rosenmüller.

Upon the left side, also, there exists a similar fleshy mass; but here it is situated at the external abdominal ring, where it forms a tumor as described above. The cord which is united to the uterus is smaller and less apparent. The testicle is a little more developed than on the opposite side. The epididymis, the ovary and the Fallopian tube present no differences.

The testicles are united to the vesiculæ seminales by the vasa deferentia. The right vas deferens is the larger and can be followed to the testicle, but the left cannot be traced. The vesiculæ seminales are situated near the neck of the bladder, in front of the vagina; the right is a little larger than the left. The ejaculatory canals probably open at the entrance of the canal of the urethra, at which point there are several small orifices; but the canal of the urethra not having been opened, it being desirable to preserve the specimen, I am not able to affirm this in a positive manner.

We find thus, two generative apparatuses, almost complete, united in the same individual; the one, male, well developed, but wanting the prostate; the other, female, presenting all the organs, but developed to a less degree than the former.

To what sex does this individual belong? To no sex probably. In reality he had testicles, which could perhaps furnish spermatozoids, notwithstanding their small size, but ejaculation being impossible, fecundation was rendered extremely difficult, not to say impossible, despite the fact that the penis was capable of an erection and even intromission; consequently this male being is condemned to impotence.

The female being possessed some of the attributes of her sex; she was able to menstruate, but the ovary is too imperfect to furnish ovules; the Fallopian tube is incapable of conducting them, and the uterus of receiving and nourishing them; moreover, the microscope does not show the presence of ovules. Consequently the female being is as impotent as the male being.

Thus, it is seen that this hermaphrodite is equally male and female, or rather, is equally impotent as male and female, incapable of reproducing with either sex, less fortunate than the vegetable, incapable of fecundating himself, though possessing two, nearly complete, generative apparatuses.

This is the complete, or nearly complete, bisexual hermaphrodite of Geoffroy Saint-Hilaire, which is worthy of being described with the cases of Marie-Madeleine, of Lefort, and the hermaphrodite of Schrell, the only known instances in the human species.

N. P—— died a bachelor, but had he any predilections for either sex ? Had he ever menstruated ? My researches in this direction are not yet completed.

We give this observation with all its details as a striking example of bisexual hermaphrodism. "These cases, moreover," said M. Tardieu, "constitute the true hermaphrodism in which the individual, possessing the attributes of both sexes, is in a state entirely incapable of forming a valid marriage ; for, whichever may be the sex to which he is united, there will be between them an identity of sex, that is to say, nullity of marriage."

